# A Rare Presentation of Synchronous Thyroglossal Cyst and Branchial Cyst in an Adult Male Patient

**DOI:** 10.7759/cureus.74630

**Published:** 2024-11-27

**Authors:** Shivashankar Ajur, Reshma Rajeev, R. N Karadi

**Affiliations:** 1 Otolaryngology - Head and Neck Surgery, Shri B.M. Patil Medical College, Hospital and Research Centre, Vijayapura, IND

**Keywords:** branchial cyst, congenital cervical masses, rare coexistence, sistrunk procedure, synchronous cyst, thyroglossal duct

## Abstract

The most common congenital cervical masses are thyroglossal cysts followed by branchial cleft anomalies. However, their synchronous presentation is uncommon. A man in his early thirties visited our ear, nose, and throat (ENT) outpatient department (OPD) with complaints of a three-month history of right-side neck swelling. On examination, a 6 cm x 4 cm non-tender, oval-shaped cystic swelling was noted towards the right side of the neck, extending obliquely to the midline. Incidentally, another swelling measuring 2 cm x 1 cm towards the left paramedian of the neck was well-defined, non-tender, cystic in consistency, and moved with deglutition and protrusion of the tongue. Investigations such as fine needle aspiration cytology, ultrasonography, and MRI neck plain and contrast confirmed the diagnosis of a right-sided type 2 branchial cyst with a coexisting thyroglossal cyst. Right branchial cyst excision with Sistrunk procedure was done under general anesthesia. This rare occurrence requires the surgeon to know the embryological basis of such cervical anomalies. We herein report a case of unusual co-occurrence of thyroglossal cyst and branchial cyst in an adult male patient.

## Introduction

Congenital neck masses are a common entity encountered in ear, nose, and throat (ENT) practice. Most of the masses are developmental anomalies arising from the branchial arches, and a smaller percentage comprises solid or cystic masses. Malignancy is very rare. Some of the most common entities encountered are thyroglossal cysts, which can develop anywhere along the thyroid gland’s developmental route and branchial cleft cysts, which most commonly arise from the second pharyngeal cleft, amongst others. However, synchronous occurrence of two different lesions is exceedingly rare, with only a few cases documented in the literature. Neck masses could further cause complications such as fistula formation and superseded infection if they go undiagnosed. This case report highlights a unique clinical presentation of a thyroglossal cyst and a branchial cyst in an adult patient, emphasizing the need for a proper understanding of developmental anatomy, early diagnosis, and surgical intervention.

## Case presentation

**Figure 1:**
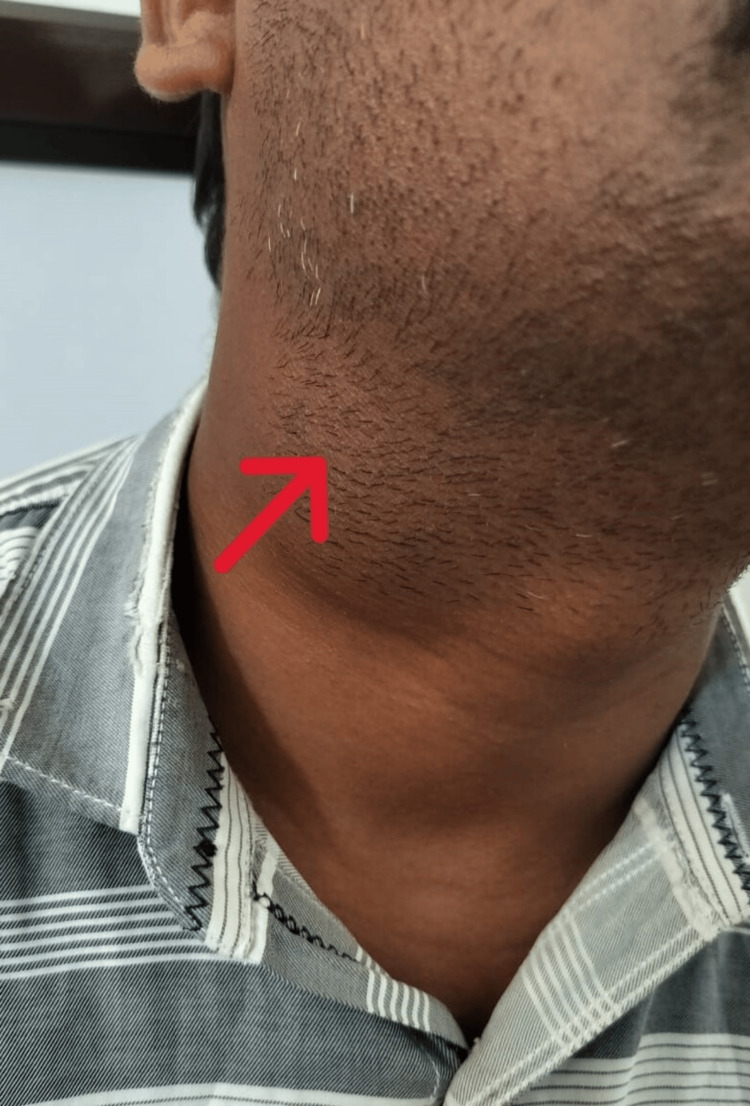
Red arrow demonstrating the branchial cyst swelling.

**Figure 2:**
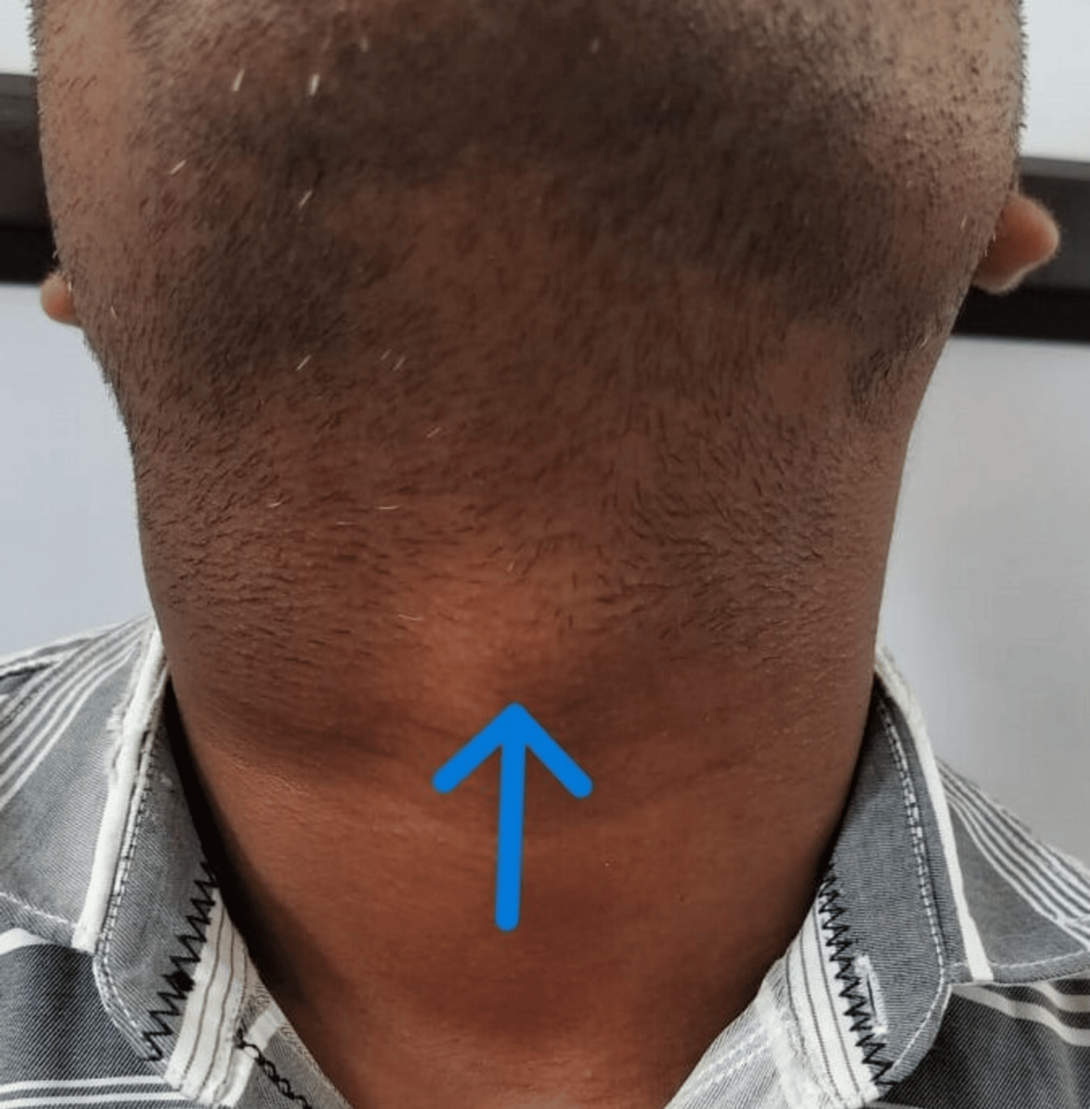
Blue arrow indicating thyroglossal cyst swelling.

**Figure 3:**
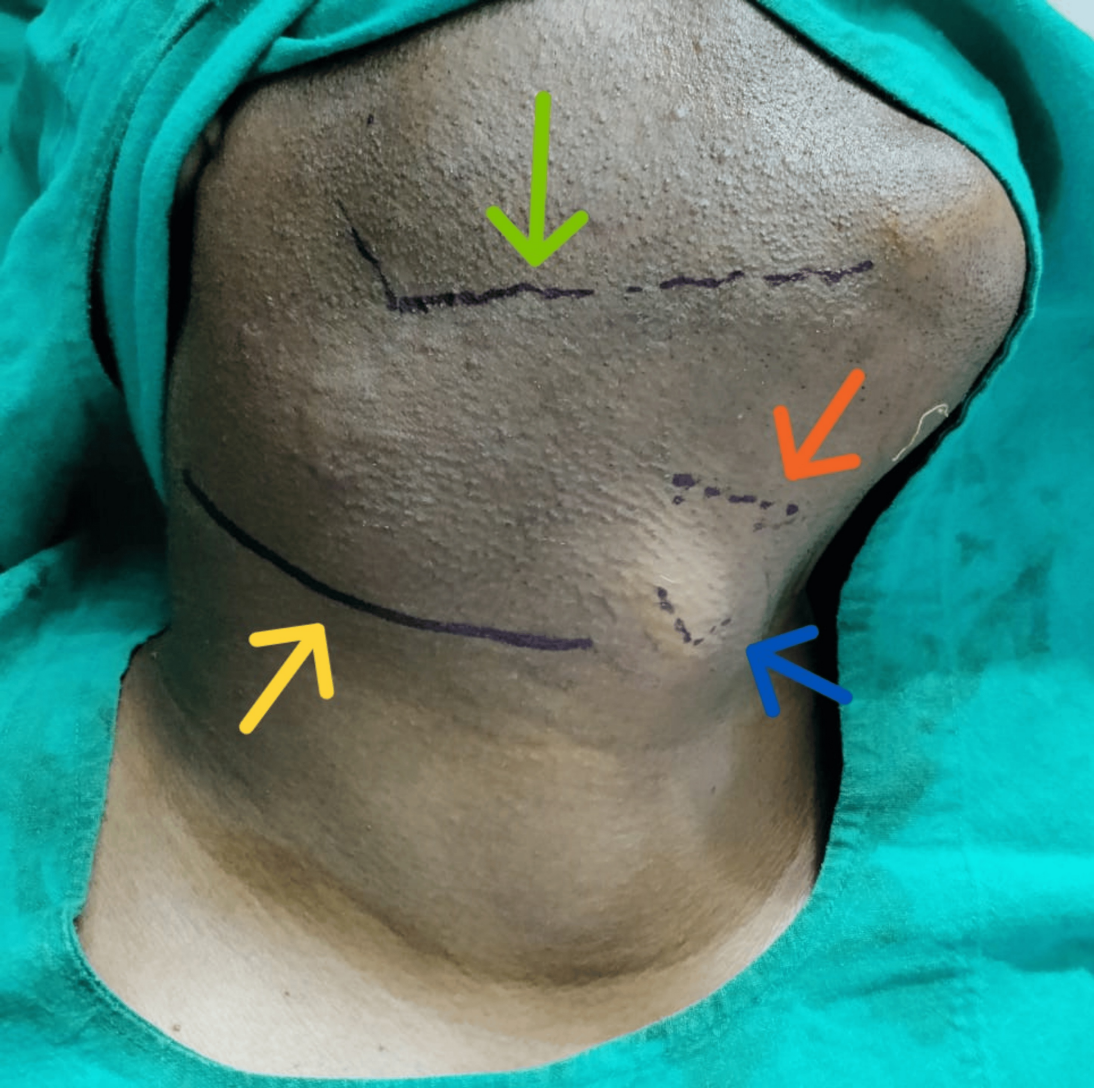
Image showing landmark. Transverse incision is indicated by yellow arrow, angle and body of mandible by green arrow, hyoid bone by orange arrow, and thyroid notch by blue arrow.

**Figure 4:**
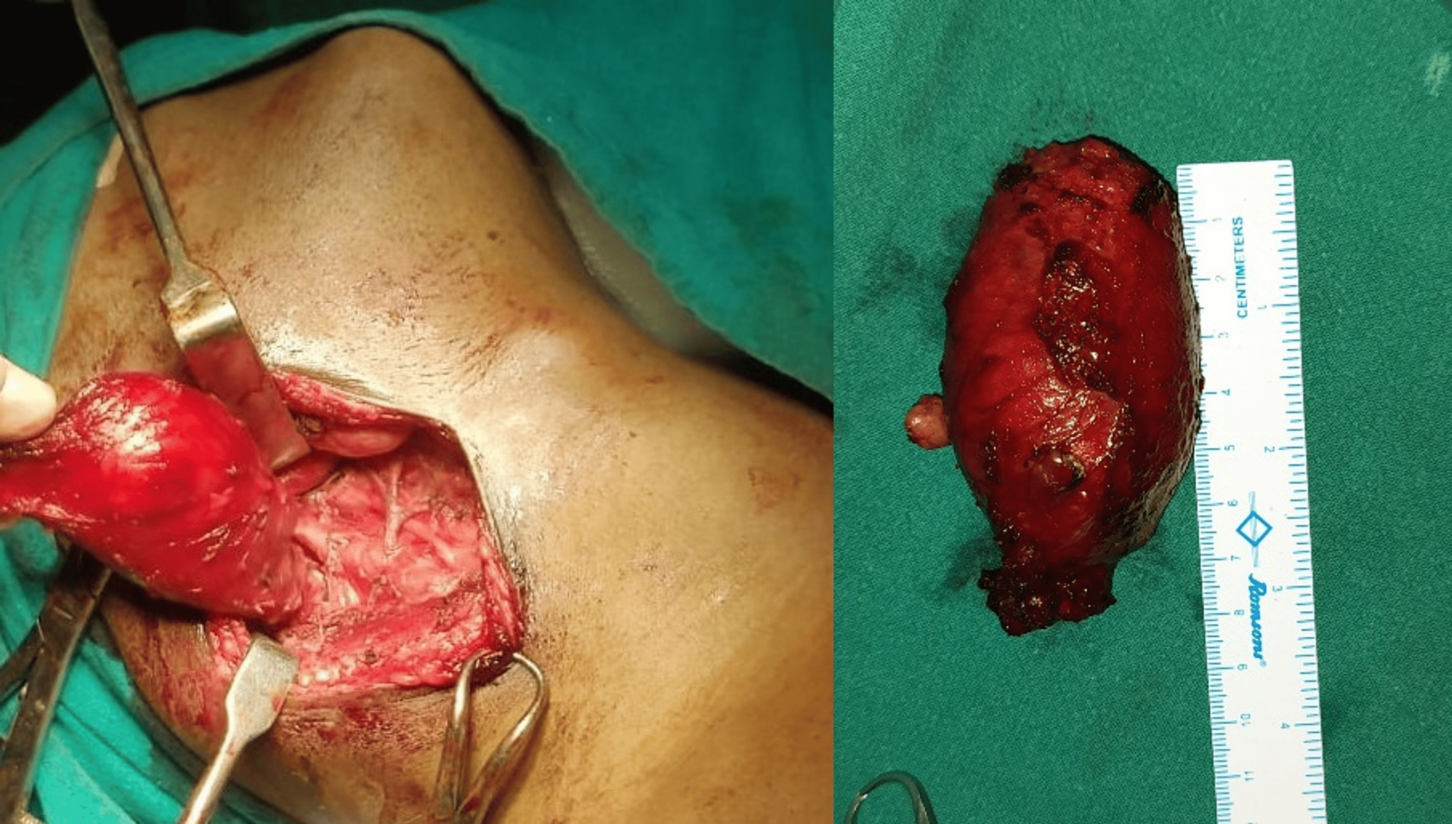
Specimen of branchial cyst for histopathological examination.

**Figure 5:**
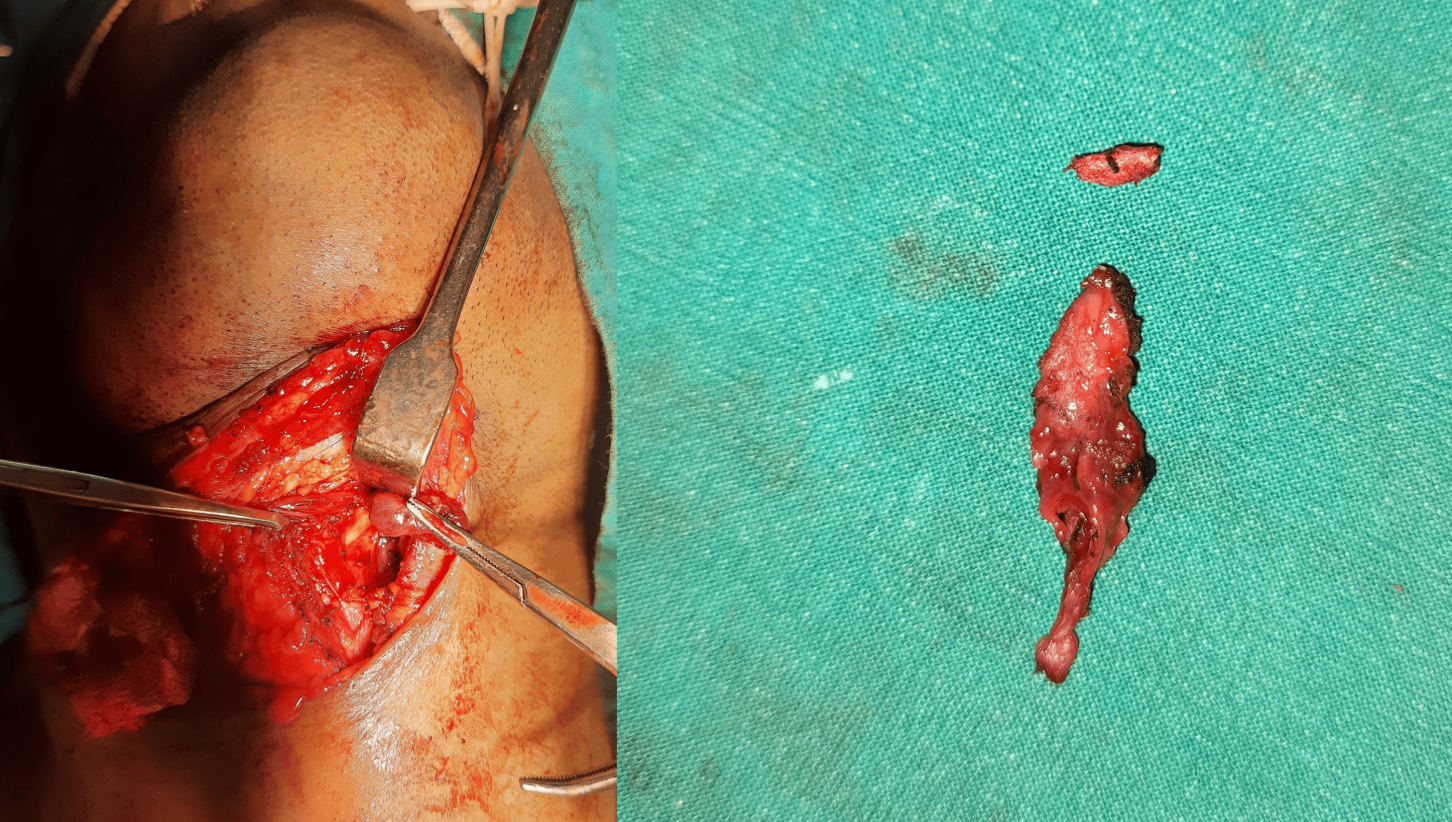
Specimen of thyroglossal cyst with tract and mid portion of hyoid bone for histopathological examination.

A 34-year-old man presented to the ENT outpatient department (OPD) with a three-month history of an oval, gradually enlarging, non-tender, soft swelling on the right side of his neck. Examination revealed a mobile, transilluminate 6 cm x 4 cm swelling (Figure 1) located below the angle of the mandible, anterior to the sternocleidomastoid muscle extending towards the midline. Incidentally, another non-tender, firm 2 cm x 1 cm swelling (Figure 2) was noted in the left paramedian aspect below the level of the hyoid bone, which moved with deglutition and protrusion of the tongue. Diagnostic imaging and fine needle aspiration cytology further confirmed the presence of a right branchial cleft cyst and a thyroglossal duct cyst. The patient underwent a Sistrunk procedure and concurrent branchial cyst excision via a single incision (Figures 3-5). He had an uneventful postoperative recovery.

**Figure 6:**
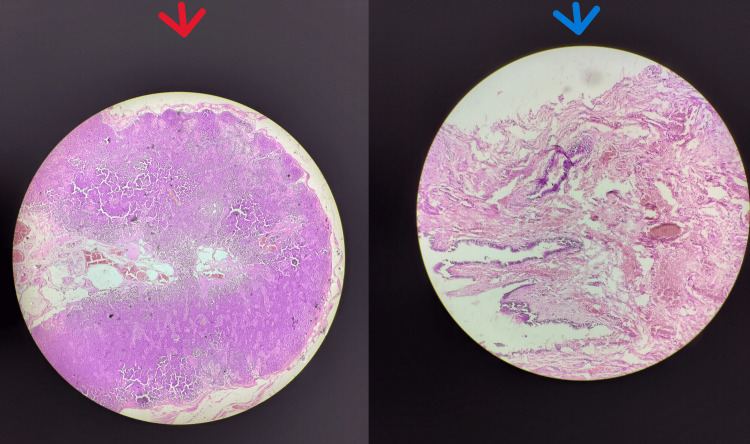
Image showing histopathological appearance of branchial cyst (red arrow) and thyroglossal cyst (blue arrow). The red arrow indicates histopathological features of the branchial cyst. Section studies reveal cyst walls lined by fibrocartilagenous tissue and abundant primary and secondary lymphoid follicles with pultaceous material within that shows scattered squamous cells, cholesterol crystals, lymphocytes, and few neutrophils in a background of proteinaceous material. The blue arrow indicates the histopathological features of the thyroglossal cyst. A smear study shows tissues lined by pseudostratified ciliated columnar epithelium. The cyst wall is fibrocartilagenous and exhibits congested and dilated blood vessels. Additionally, foci of normal thyroid follicles are noted.

Histopathological examination confirmed the swelling to be a branchial cyst and thyroglossal cyst, respectively (Figure 6).

## Discussion

The literature contains only a handful of reports of synchronous thyroglossal and branchial malformations, with the same occurring in adults being even rarer. Case reports by Alarfaj et al. and Sima et al. were instances where adult patients presented with neck swellings that were diagnosed to be synchronous thyroglossal and branchial cysts [1,2]. Both cases had a gradual and asymptomatic progression of the neck swelling without pain or significant inflammation with a similar surgical management that entailed a lateral transverse neck incision extended anteriorly to remove both swellings in a single sitting.

In other studies, for instance, Jaswal et al. in 2008, reported a case of a 13-year-old girl with a complete branchial and thyroglossal fistula at the same time. This was confirmed by radiological evidence on a fistulogram [3]. Following this, Kyi et al. reported a case of a 44-year-old man in 2012 with a history of recurrent discharge from an opening in the right lower neck for four years. He was diagnosed with a second branchial cleft fistula and a thyroglossal duct cyst [4]. Both these cases involved fistulas (branchial and thyroglossal) and recurrent discharge, indicating a similar symptomatic presentation despite the differences in the patients' ages. Both cases were managed with the same approach. A classical Sistrunk operation was performed for the thyroglossal fistula, and for the branchial fistula, the fistulous tract was excised using a step ladder approach, with the fistula excised entirely up to the tonsillar fossa and the end ligated with silk.

Similarly, in 2016, Lee et al. described a 34-year-old male presenting with recurrent anterior and lateral neck swelling with mucoid discharge since childhood, diagnosed with a left second branchial cleft fistula and a likely thyroglossal duct cyst [5]. This case was characterized by chronic recurrent mucoid discharge, a history of bilateral tonsillectomy, and large cystic lesions identified on CT imaging, differentiating it from other cases primarily regarding the chronicity and imaging findings. In another case by Mahdoufi et al. in 2016, an eight-year-old girl with recurrent lateral cervical discharge from a fistula since birth and a lump on the upper neck for six months was diagnosed as a thyroglossal cyst and a fistulous branchial cyst [6]. The presentation involved a congenital fistula with discharge and a newly developing lump, highlighting the combination of chronic congenital anomalies with an acute increase in the mass size, differing from other cases that did not emphasize such acute changes.

Of all similar presentations, five cases, including ours, involved adults. Meanwhile, only two instances involved children. There are several unique aspects to our case. Firstly, it is only the third documented instance of an adult with a coexisting thyroglossal duct cyst and a second branchial cleft cyst. This emphasizes that these anomalies, typically seen in younger patients, can also present in adults. As such, the possibility of concurrent thyroglossal duct and branchial cleft cysts should be included in the differential diagnosis for neck lesions in adults. Secondly, the patient had no prior history of infection or surgery for congenital neck anomalies. This suggests that asymptomatic thyroglossal duct and branchial cleft cysts can exist as undetectable neck lesions in adults. Lastly, surgical management using the same incision to address both pathologies results in better cosmesis and better patient compliance and is more cost-effective.

## Conclusions

This rare occurrence highlights the importance of knowing the embryological basis of such cervical anomalies, enabling surgeons to be vigilant in diagnosis and avoiding unexpected intraoperative scenarios. Removal of both pathologies in one surgery benefits patients by saving time and also offers better cosmetic benefits.
